# Photoinduced Hydrogel-Forming Caged Peptides with Improved Solubility

**DOI:** 10.1021/acsomega.3c08289

**Published:** 2024-01-30

**Authors:** Kata N. Enyedi, Bettina Basa, Gábor Mező, Eszter Lajkó

**Affiliations:** † Faculty of Science, Institute of Chemistry, Department of Organic Chemistry, Eötvös Loránd University, Pázmány Péter sétány 1/A, 1117 Budapest, Hungary; ‡ HUN-REN-ELTE Research Group of Peptide Chemistry, Eötvös Loránd University, Pázmány Péter sétány 1/A, 1117 Budapest, Hungary; § Department of Genetics, Cell- and Immunobiology, Semmelweis University, Nagyvárad tér 4, 1089 Budapest, Hungary

## Abstract

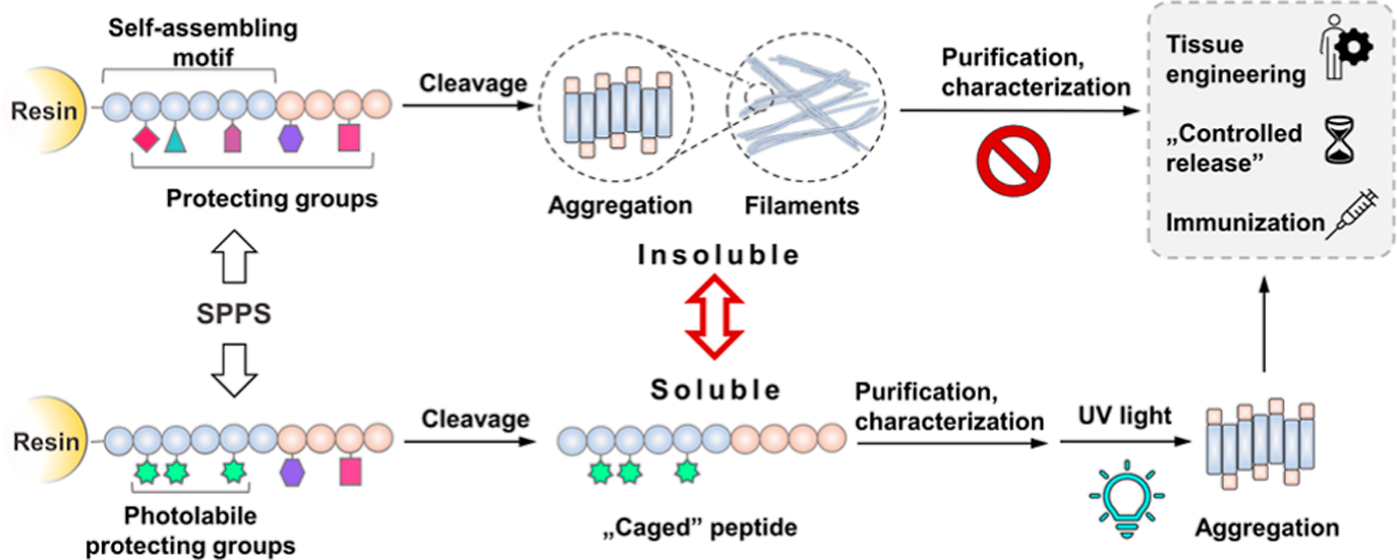

Self-assembling peptides are attractive alternatives in the field of biomaterial science due to their variability and biocompatibility. Unfortunately, such peptides have poor solubility, and their purification, synthesis, and overall handling are challenging. Our main objective was to develop a cage peptide design with full control over self-assembly. Theoretically, aggregation can be suppressed by temporally masking the amino acid side chains at critical positions. Taking into account several biological and synthetic requirements, a photosensitive protecting group, *p*-hydroxy-phenacyl (pHP), was chosen as the “masking” moiety. To test our theory, EAK16-II was chosen as a model self-assembling peptide, and a caged derivative containing photosensitive pHP groups was synthesized. Both spectroscopic and in vitro experiments on A2058 melanoma cells confirmed our hypothesis that the caged-EAK16-II peptide has good solubility and that the hydrogel formed after photolysis results in similar viability and cell aggregate formation of melanoma cells as the native EAK16-II-based hydrogel.

## 1 Introduction

Peptide-based hydrogels are one of the most current fields of material science and pharmaceutical research due to their high biocompatibility and wide range of applications (e.g., tissue engineering, controlled drug release, and immunization).^1,2^

In the production of cross-linked peptide networks, usually nonpeptide cross-linking structures are used, which can influence the biological properties of the peptide-based hydrogel.^3,4^ However, self-assembling peptides can be an alternative as they can be organized into well-characterized and biocompatible hydrogels. Unfortunately, the practical use of such peptides is hampered by their fundamental aggregation property. This makes their synthesis, handling, and especially their purification challenging. To overcome this, we designed a caged peptide approach (covalent linkage of a photolabile protecting group) that enables the practical production of self-assembling peptide hydrogels.

We hypothesized that self-assembly could be suppressed by using a suitable protecting group at the aggregation site of the peptide. However, to induce aggregation in a controlled manner, the protecting group should be selectively removed. In our design, a photolabile protecting group was chosen to avoid the use of additional chemicals that are not necessarily biocompatible. To be used effectively, such a protecting group must also adhere to certain specific criteria. Considering the synthetic and biological aspects, the requirements include: (i) good solubility in aqueous and buffer media, (ii) steric hindrance to interfere with aggregation, (iii) a biocompatible photocleaved metabolite, and (iv) compatibility with solid phase peptide synthesis (SPPS).

To meet several of these criteria, we chose *p*-hydroxy-phenacyl (pHP) as our photosensitive protecting group. It is water-soluble and bulky, and its photocleaved metabolite (*p*-hydroxyphenylacetic acid, HPAA) is an intrinsic compound (a tyrosine metabolite).^5^ However, its use in SPPS is highly problematic due to its unprotected hydroxyl group, which can lead to several side reactions (e.g., N–O acyl transfer) during peptide synthesis.^6,7^ Therefore, we aimed to synthesize a *tert*-butyl (^*t*^Bu) protected pHP-derivative, to be utilized as a “two steps” protecting group in SPPS.

Previously, the pHP group was reported primarily as a carboxylic acid-protecting group. However, due to the fact that the most popular self-assembling peptides are counterion based (e.g., lysin-glutamic acid), we synthesized Fmoc-l-Lys(pHP(^*t*^Bu))–OH. Here, we demonstrated that, under the appropriate conditions, this group can also be used for amine protection contrary to previous a report.^8^

To evaluate our concept, a known hydrogel-forming peptide EAK-16-II (Ac-AEAEAKAKAEAEAKAK-NH_2_) was chosen.^9,10^ As the self-assembly ability of this peptide is based on the strong ionic interactions between the oppositely charged amino-acid side chains, it proved to be an ideal model.^11^ As this peptide has the potential for use as a biomaterial, it also provided us the opportunity to test the effectivity and applicability of our design in a biological system.^12,13^ Therefore, our caged peptide was used for the formation of an in vitro 3D cell culture. This experiment also verified our assumption that the protecting group is fully biocompatible and that self-assembly can be triggered by removing the pHP protecting group.

## 2 Experimental Section

### 2.1 Materials

4-*tert*-Butoxystyrene and *tert*-butyl hydroperoxide (TBHP) were purchased from Sigma-Aldrich (St. Louis, MO, USA). Acetonitrile, dimethyl sulfoxide (DMSO), methyl *tert*-butyl ether (MTBE), iodine, *n*-hexane, ethyl-acetate, MeTHF, and NaNO_2_ were purchased from VWR International Ltd. (Debrecen, Hungary).

All reagents used in peptide synthesis were purchased from Iris Biotech GmbH (Marktredwitz, Germany), unless otherwise stated. All of the reagents and solvents were used without further purification.

### 2.2 Characterization

NMR spectra were recorded using a Bruker Ascend 400 spectrometer (Billerica, MA, USA). Chemical shifts (δ) are reported in parts per million (ppm). Signals are indicated as s (singlet), d (doublet), t (triplet), q (quartet), dt (double triplet), and m (multiplet).

MS spectra were recorded using the LC–MS technique on an Q-TOF instrument (Waters Corporation, Milford, MA, USA). LC was performed using a Waters Acquity UPLC BEH C18 1.7 μm column (2.1 × 50 mm) (Waters Corporation, Milford, MA, USA), at 25 °C with linear gradient elution (0 min 2% B, 1 min 2% B, 17 min 100% B, 17.5 min 100% B, 18 min 2% B, 21 min 2% B) with eluent A (0.1% formic acid in water) and eluent B [0.1% formic acid in MeCN–H_2_O (80:20, v/v)] at a flow rate of 300 μL/min.

Analytical thin-layer chromatography (TLC) was carried out on Merck DC precoated TLC plates (Merck, Darmstadt, Germany) with 0.25 mm Kieselgel 60 F254. Visualization was performed by fluorescence quenching under 254 nm irradiation.

Analytical RP-HPLC was conducted on a KNAUER 2.1 S HPLC system (KNAUER, Bad Homburg, Germany) using a Macherey Nagel Nucleosil 100–5C18 column (250 × 4.6 mm) with 5 μm silica as the stationary phase. Eluents A [dH_2_O/0.1% trifluoroacetic acid (TFA)] and B [0.1% TFA in MeCN–H_2_O (80:20, v/v)] were used for linear gradient elution (0 min 2% B; 27 min 100% B). Eluents were applied at a flow rate of 1 mL/min, and peaks were detected at 220 nm.

Transmittance was measured using an UV–vis spectrophotometer (Jasco V-650, Jasco, Cremella, Italy) in phosphate-buffered saline (PBS, pH 7.4, 1×) buffer.

Dynamic light scattering (DLS) measurements were carried out on a Nanolab 3D instrument (LS Instruments, Switzerland) in PBS buffer.

### 2.3 Synthesis

#### 2.3.1 Synthesis of 1-[4-(*tert*-Butoxy)phenyl]-2-iodoethan-1-ol (Compound **2**)

4-*tert*-Butoxystyrene (2 mL, 10.6 mmol, 1 equiv) was dissolved in 40 mL of MeCN/dH_2_O (8:2). First DMSO was added (1.5 mL, 21.2 mmol, 2 equiv), followed by iodine (2.7 g, 10.6 mmol, 1 equiv), and TBHP (70 wt % in H_2_O, 3 mL, 21.2 mmol, 2 equiv) (following the sequence of chemicals added is recommended to reduce the formation of other byproducts). The reaction was stirred at room temperature and was monitored using TLC. When all the starting material had reacted (∼2–2.5 h), saturated ascorbic acid solution (in H_2_O) was added until the reaction mixture lost its yellow color. This was immediately followed by the addition of NaHCO_3_, until the reaction mixture reached pH 8. The solution was extracted with *n*-hexane three times, and the organic layer was evaporated. Without further purification, it was directly used in the next step.

Rf: 0.4 [in *n*-hexane/ethyl-acetate (3:1)].

^1^H NMR (400 MHz, CDCl_3_ with 0.05% TMS): δ 7.28 (d, 2H, *J* = 8.0 Hz), 6.98 (d, 2H, *J* = 8.0 Hz), 4.83 (dd, 1H, *J* = 2.8, 7.2 Hz), 3.48 (dd, 1H, *J* = 2.4, 8.0 Hz), 3.40 (dd, 1H, *J* = 8.0, 8.0 Hz), 1.36 (s, 9H).

#### 2.3.2 Synthesis of 1-[4-(*tert*-Butoxy)phenyl]-2-iodoethan-1-one (Compound **3**)

Compound **2** (2.5 g, 7.8 mmol, 1 equiv) was diluted with MeTHF (5 mL), and cooled Jones-reagent (10 mL, 2 equiv) was added dropwise at room temperature under intense stirring and was monitored using TLC. When all the starting material had reacted (∼1–1.5 h), the solution was extracted with *n*-hexane three times. The organic layer was washed with water three times, then evaporated, and without further purification, was directly used in the next step.

Rf: 0.6; *n*-hexane/ethyl-acetate (3:1).

^1^H NMR (400 MHz, CDCl_3_ with 0.05% TMS): δ 7.92 (d, 2H, *J* = 8.0 Hz), 7.03 (d, 2H, *J* = 8.0 Hz), 4.32 (s, 2H), 1.44 (s, 9H).

#### 2.3.3 Synthesis of 1-[4-(*tert*-Butoxy)phenyl]-2-hydroxyethan-1-one (Compound **4**)

Compound **3** (2 g, 6.2 mmol, 1 equiv) was diluted with 68 mL of ice-cold DMF, and under continuous stirring, NaNO_2_ (0.52 g, 7.5 mmol, 1.2 equiv) was added. The reaction mixture was allowed to warm up to room temperature (∼2 h) and was followed by TLC. When all of the starting material had reacted, the solvent was evaporated. The residue was extracted with water and *n*-hexane three times, and the organic layer was separated, dried over Na_2_SO_4_, and evaporated.

Rf: 0.1; *n*-hexane/ethyl-acetate (3:1).

^1^H NMR (400 MHz, CDCl_3_ with 0.05% TMS): δ 7.86 (d, 2H, *J* = 8.0 Hz), 7.06 (d, 2H, *J* = 8.0 Hz), 4.83 (s, 2H), 1.45 (s, 9H).

#### 2.3.4 Synthesis of Compound **5** and Compound **6**

The synthesis was performed based on the previously described method by Katayama.^8^ Compound **4** (0.5 g, 2.4 mmol, 1 equiv), disuccinimidyl carbonate (0.677 g, 2.6 mmol, 1.1 equiv), 4-dimethylaminopyridine (32 mg, 0.26 mmol, 0.1 equiv), and *N*,*N*-diisopropylethylamine (1.255 mL, 7.2 mmol, 3 equiv) were dissolved in dry DMF (14 mL) and stirred for 1 h at room temperature in a flask protected from light (Compound **5**).

To this mixture, Fmoc-Lys-OH hydrochloride (0.89 g, 2.4 mmol, 1 equiv) was added and was stirred overnight (Compound **6**). The solvent was evaporated, and the residue was extracted with 1 M HCl and DCM. Then, it was washed with brine, and the organic phase was dried over Na_2_SO_4_. After filtration and evaporation, the residue was chromatographed on silica-gel with toluene/EtOAc/AcOH (50:50:1). Analytical measurements were conducted using RP-HPLC, LC–MS, and ^1^H NMR spectroscopy. The analytical HPLC chromatogram and MS spectrum of Compound **6** are shown in Supporting Information (Figure S1A,B).

^1^H NMR (400 MHz, CDCl_3_ with 0.05% TMS): δ 7.79 (m, 2H), 7.74 (m, 2H), 7.58 (m, 2H), 7.39 (m, 2H), 7.29 (m, 2H), 7.0 (m, 2H), 5.83 (d, 1H, *J* = 8.0 Hz), 5.34 (m, 1H), 5.19 (m, 2H), 4.37–4.32 (m, 3H), 4.19 (t, 1H, *J* = 6.76, 5.48 Hz), 3.23 (m, 2H), 1.89 (d, 2H, *J* = 8.0 Hz), 1.57 (m, 2H), 1.38 (m, 11H) (Figure S2).

#### 2.3.5 Peptide Synthesis

Peptides were synthesized manually, using the Fmoc/^*t*^Bu strategy on Fmoc-Rink Amide MBHA resin (0.6 mmol/g loading capacity). Couplings were carried out using diisopropylcarbodiimide/HOBt in DMF (3 equiv compared to resin capacity).

The protocol of the Fmoc/^*t*^Bu strategy was as follows: (i) washing with DMF (3 × 0.5 min), (ii) Fmoc deprotection with 2% DBU, 2% piperidine in DMF (2 × 2 min, 1 × 5 min, 10 min), (iii) washing with DMF (4 × 0.5 min), (iv) Fmoc-protected amino acid derivative coupling: DIC: HOBt in DMF (3 equiv each) (1 × 45 min), (v) washing with DMF (2 × 0.5 min), (vi) washing with DCM, and (vii) ninhydrin assay.

The completed peptides were removed from the resin using 10 mL of the cleavage mixture (95% TFA and 5% dH_2_O) and stirred for 1.5 h at 0 °C. Resins were filtrated, and the crude products were precipitated in ice cold MTBE (methyl-*tert*-butyl ether) and centrifuged for 4 min at 4000 rpm. The crude products were dissolved in acetonitrile/water, and the solution was lyophilized and purified by RP-HPLC and ultimately characterized by means of analytical RP-HPLC and LC–MS (Figure S3A,B).

### 2.4 Cell Culturing

Human melanoma cell line A2058 was obtained from the European Collection of Authenticated Cell Cultures (ECACC, Salisbury, UK) and was cultured in RPMI 1640 medium supplemented with 10% (v/v %) fetal bovine serum (Biosera Europe, Nuaillé, France), 1% l-glutamine, and 1% penicillin/streptomycin (Gibco, Invitrogen Corporation, New York, USA). Cells were propagated in standard cell culture conditions (37 °C, 5% CO_2_).

### 2.5 Preparation of Hydrogels for In Vitro Measurements

Peptides were dissolved in PBS (1×) at a 1.2 mM concentration. 50–50 μL of the resulting stock solution was added to a 96-well, optical bottom plate. The plate was illuminated with an UV lamp (Sylvania 254 nm, 8 W) for 20 min, followed by the distribution of 50–50 μL of RPMI-1640 medium [supplemented with 10% (v/v %) FBS and 1% l-glutamine, 1% penicillin/streptomycin] into the wells. Then, the plate was incubated for 1 h.

### 2.6 Viability Assay

For viability experiments, after hydrogel formation or in 2D, A2058 cells were seeded at a density of 10,000 cells/well in 100–100 μL of serum containing medium on a 96-well plate with optical bottom and were then incubated for 96 h.

Viability tests were performed using CellTiterGlo (Promega, Madison, WI, USA) reagent to determine the quantity of viable cells in both 2D setups and in hydrogels. For this, 200–200 μL of the CellTiterGlo reagent was added to each well, and the plate was kept in dark at room temperature for 30 min. The luminescence signal was measured by a Fluoroskan FL microplate fluorometer and luminometer (Thermo Scientific, Waltham, MA, USA). For each assay, triplicates were used. All quantitative data are presented as the mean ± standard deviation (SD). The results were evaluated with MS Excel and OriginPro 2020 software (OriginLab Corporation, Northampton, MA, USA).

### 2.7 Fluorescence Microscopic Detection

For the 3D microscopic imaging of the hydrogels, cells were treated with acridine orange (at 0.1 mM) and incubated for 30 min. Fluorescence measurements were carried out on a Zeiss Celldiscoverer 7 (Zeiss, Jena, Germany) using 10× magnification (Plan-Apochromat λ/0.35 NA objective with 2× tube lens) and acquisition of *Z*-stacks with 10 μm intervals between individual planes. The images were processed by using ZEN Blue 2.6 software (Carl Zeiss AG, Jena, Germany).

### 2.8 Statistical Analysis

Results were evaluated using MS Excel and OriginPro 2020 software (OriginLab Corp., Northampton, MA, USA). Data are presented as the mean ± standard deviation (SD) (*n* = 3). Data were statistically analyzed using one-way analysis of variance followed by Tukey’s posthoc test. The viability of A2058 cells growing in each hydrogel was compared to the control cells growing in 2D (normalized live cells = 100%).

## 3 Results and Discussion

### 3.1 Synthesis of Photosensitive Amino Acid Derivative

In our work, we developed a photolabile pHP derivative that can be used as a protecting group for peptide side chains in order to improve the handling of aggregating peptides.

**Scheme 1:**
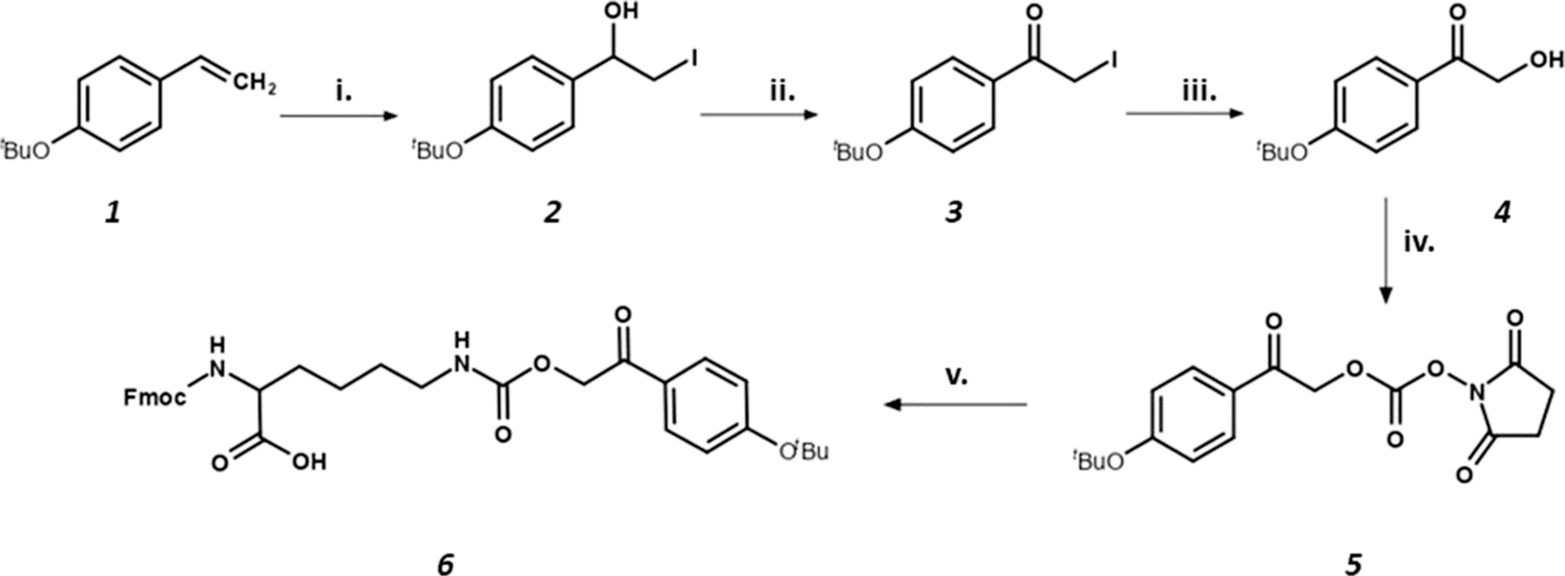
Synthesis of Fmoc-l-Lys(pHP(*t*Bu))–OH (i) I_2_, TBHP, DMSO in H_2_O/MeCN, 1.5 h; (ii) Na_2_Cr_2_O_7_/H_2_SO_4_ in MeTHF, 30 min; (iii) Na_2_NO_2_ in DMF, 30 min, 0 °C; (iv) DSC, DIPEA in DMF, 3 h, (v) Fmoc-l-Lys-OH, DIPEA in DMF.

The first step was the synthesis of the *^t^*Bu-protected pHP derivative, which counteracts the potential side reactions that can occur during SPPS, thereby allowing its utilization as an amino acid side-chain protecting group. The three-step reaction showed high efficiency, requiring no additional purification to the final product, and was also found to be scalable up to a few grams (Scheme 1). The resulting pHP(^*t*^Bu) (Compound **4**) was then further used as a carbamate type protection for the lysine ε-amine (Compound **6**, Figures S1 and S2), and later it was investigated in SPPS to prepare the caged peptide.

### 3.2 Peptide Design and Characterization

Our model peptide EAK16-II (Ac-AEAEAKAKAEAEAKAK-NH_2_) is one of the first known self-assembling peptides. It forms fibrillar structures that organize into high water content hydrogels.^10^ Its aggregation is based on the presence of opposite charges (lysine–glutamic acid) and is triggered by exiguous amount of mono- or bivalent cations (e.g., Na^+^ and Ca^2+^). Therefore, its handling (e.g., synthesis, conjugation, and purification) and biological application can be challenging.^9,14^

**Scheme 2:**
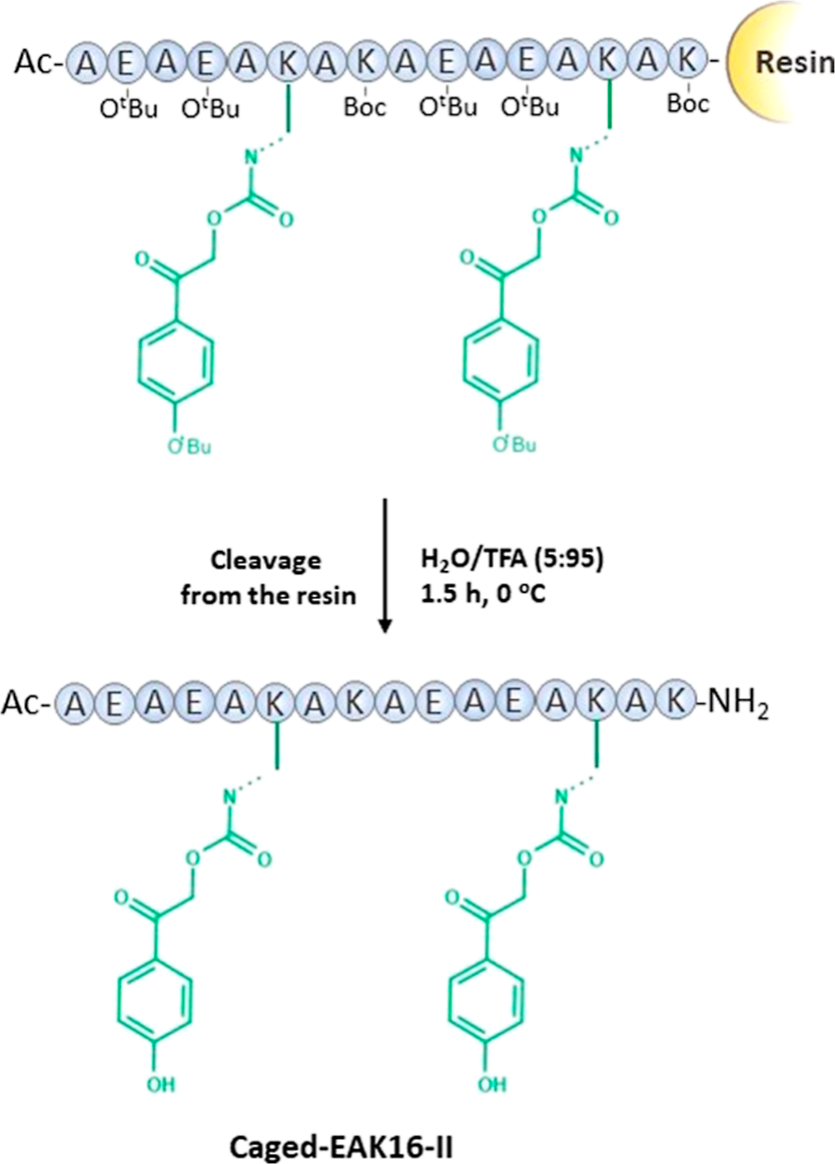
Scheme of Caged Peptide Synthesis with a “Two-Step” pHP Protection

To temporarily suppress this feature, the photolabile pHP protecting group was used at two points in the peptide sequence (Lys^6^ and Lys^14^) (Scheme 2). This was achieved using the synthesized Fmoc-l-Lys(pHP(^*t*^Bu))–OH derivative. The photolabile protecting group pHP was found to be compatible with the conditions of SPPS. After the cleavage of the peptide from the resin, it remained on the ε-amines of the lysine side chains, which resulted in a semiprotected, caged peptide (Scheme 2). HPLC and LC–MS analysis demonstrated that all compounds, including photosensitive amino acid derivative (Figure S1) and peptides (Figure S3) were >95% pure.

**Figure 1:**
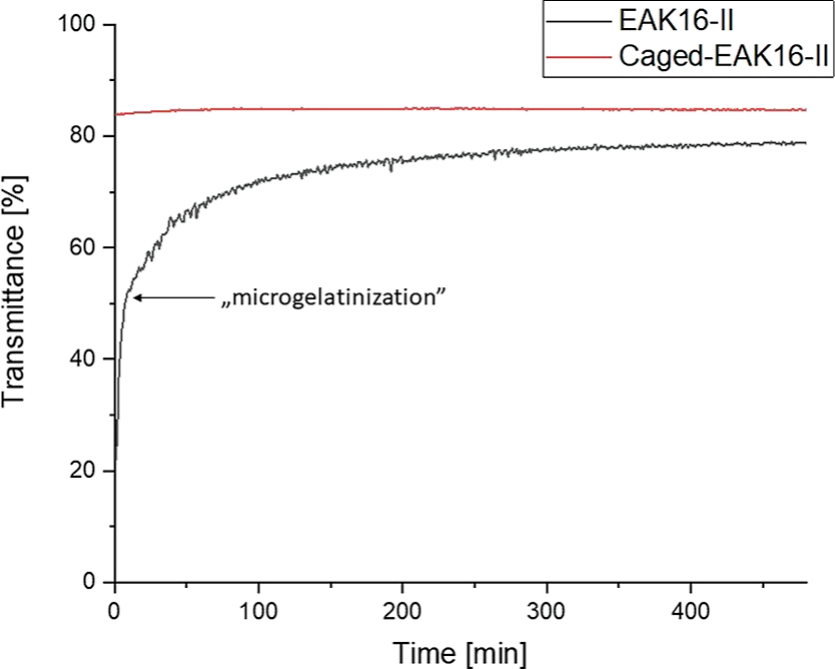
Optical transmittance of native (nonprotected) EAK16-II peptide and caged-EAK16-II (300 μM) in PBS, 25 °C, 532 nm, 8 h.

The incorporation of the pHP group significantly improved the solubility and the handling of the peptide as no interference with solubility and no difficulties during HPLC purification were observed (Figure S3). To support this change in “behavior” of the caged derivative, spectroscopic (transmittance) measurements were conducted, which confirmed the suppressed aggregation compared to the nonprotected peptide (Figure 1).

Around 10 min after the starting point, the native EAK16-II showed “microgelatinization” (sedimentation of peptide aggregates), which led to an increase in transmittance. In contrast, the caged peptide exhibited a constant transmittance (∼85%) over a time period of 8 h (Figure 1). These turbidity measurements were conducted at a 300 μM concentration, which was also used for in vitro experiments. These results indicated that the caged peptide could not aggregate even above the critical aggregating concentration (0.1 mg/mL = ∼ 60 μM)^14^ of the native peptide.

In the caged peptide-derivative, pHP groups temporarily mask the lysine side chains and suppress the ionic interactions. In addition, pHP is a bulky protecting group, which contributes even more to this masking property. However, pHP is quite hydrophilic; thus, it does not cause the precipitation of the peptide in aqueous media. As a result, when caged peptides are used, there is no driving force for fibril (and hydrogel) formation.

**Figure 2:**
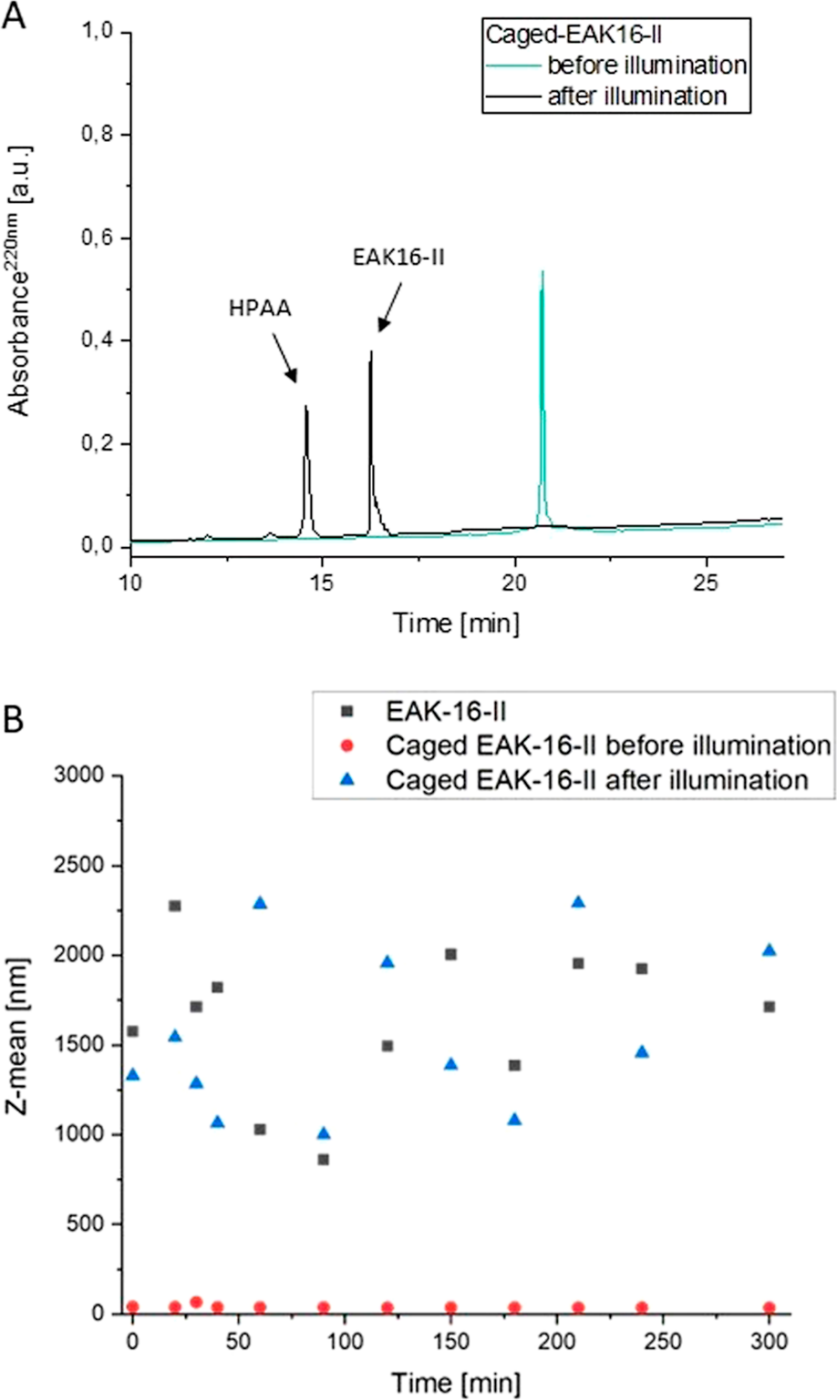
(A) Analytical RP-HPLC of caged peptide (caged-EAK16-II) before and after photolysis (illumination). Peaks were detected at 220 nm, and the peptide solutions (300 μM) in PBS were illuminated at 254 nm for 15 min. (B) Size of aggregates of native (nonprotected) EAK16-II peptide and caged-EAK16-II (60 μM) by dynamic light scattering (DLS) measurement (at 25 °C with 532 nm for 5 h) before and after illumination in PBS.

The caged peptide was deprotected using UV, and the release of the native EAK16-II peptide and HPAA was verified through RP-HPLC (Figure 2A), which is consistent with previous reports.^15,16^ Additionally, DLS measurements (at 25 °C with 532 nm at 5 h) also confirmed the self-assembling property of caged-EAK16-II after illumination (Figures 2B and S4).

### 3.3 In Vitro Application

To test our caged peptide design in a biological in vitro setting, we exploited the main characteristic of the EAK16-II peptide, its hydrogel-formation ability, to prepare 3D cell cultures. We chose the A2058 human melanoma cell line as our model cell. In this setting, our goal was simply to demonstrate that by regulating the self-assembling property of a peptide, we can create a peptide-hydrogel system identical to the original (nonprotected) hydrogel via photolysis of a caged derivative. By using such an in vitro system, we can also confirm the practical applicability and usability of our idea.

**Figure 3:**
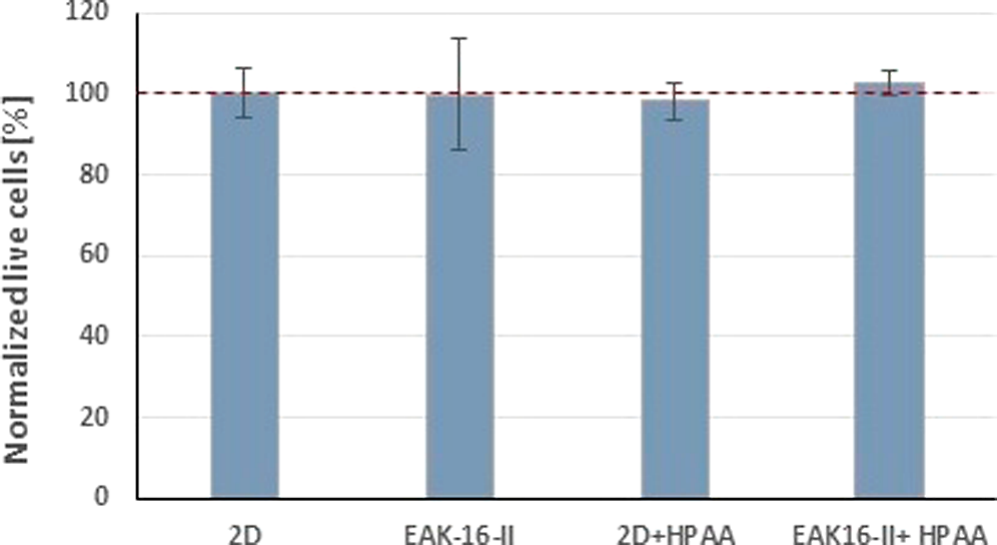
In vitro cytotoxicity of *p*-hydroxyphenylacetic acid (HPAA, 1 mM) on A2058 human melanoma cell line growing in EAK16-II (300 μM) hydrogel (3D) or in a monolayer (2D) for 96 h. Cell viability measurements were performed using CellTiterGlo assay (Promega, Madison, WI, USA). Viability data were compared and normalized to 2D growing control cells (normalized live cells = 100%). Data are presented as mean values ± standard deviation (SD) from three independent experiments.

It was an important element of our design that the metabolite of the photosensitive group would not affect any biological system. As a first step, the cytotoxicity of the released metabolite (HPAA) was tested in a classical 2D system (plastic surface of cultureware) as well as in the original EAK16-II-hydrogel. In both cases, HPAA did not have any long-term toxic effect (Figure 3).

To test the biocompatibility and applicability of our caged peptide in vitro, hydrogels were prepared from both native EAK16-II and its caged peptide derivative. After the gel formation, A2058 cells were loaded onto it and were cultured for 96 h. We aimed to show that hydrogels formed from EAK16-II and photolyzed/released caged peptide behaved identically even for a longer incubation time. After 96 h incubation, it was shown that A2058 cells created distinct cellular aggregates in both 3D hydrogels in contrast to the 2D control. The 2D cell culture already reached confluence at this point, and therefore, longer-term culturing was not conducted.

**Figure 4:**
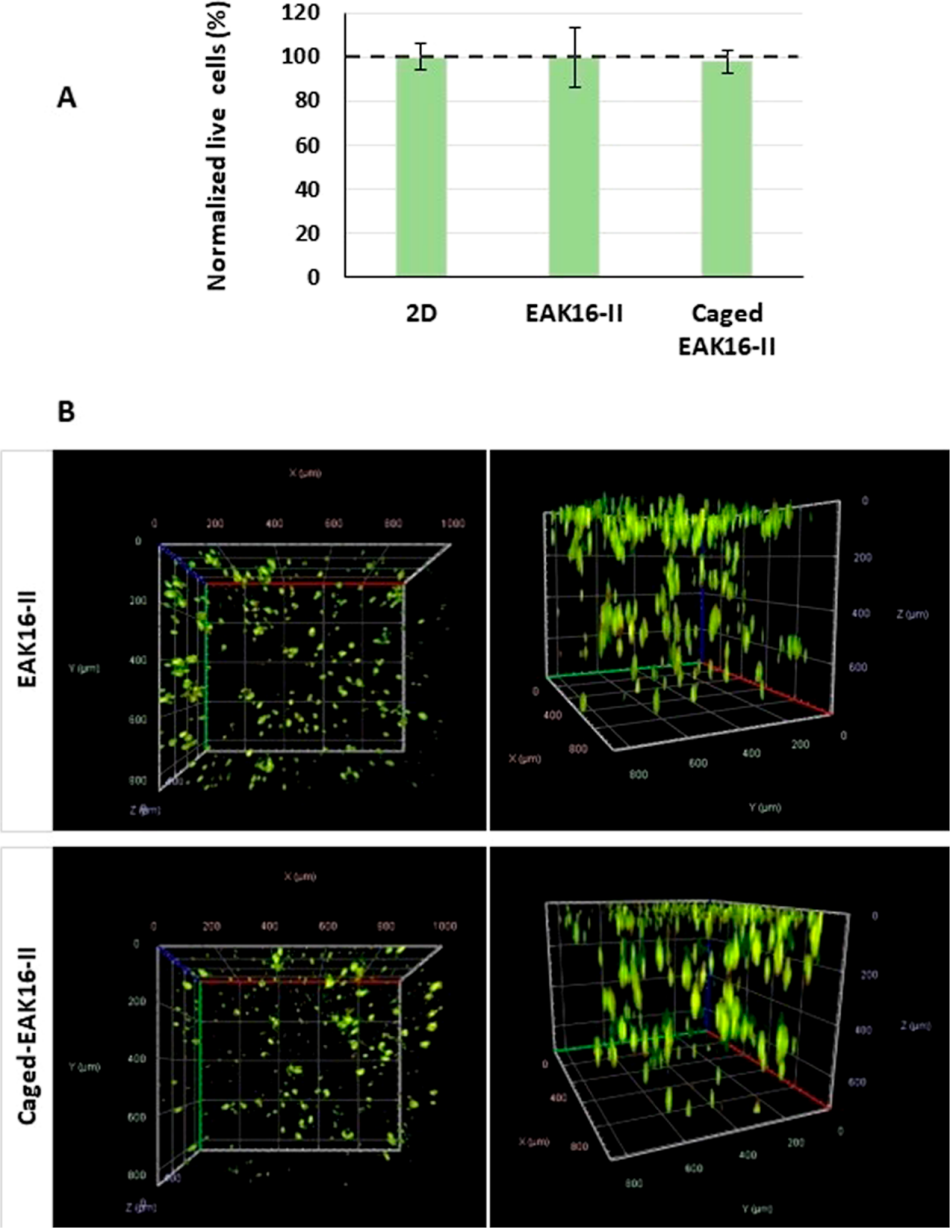
(A) Viability of the A2058 human melanoma cell line in native EAK-16-II and caged peptide hydrogels as well as in monolayer (2D) after 96 h. Cell viability was measured using CellTiterGlo assay (Promega, Madison, WI, USA) and normalized to 2D growing cells (control cells) (normalized live cells = 100%). Data are presented as mean values ± standard deviation (SD) from three independent experiments. (B) 3D representation of cellular arrangements in both types of hydrogels. Cells were stained with orange acridine (0.1 mM). The cellular aggregates were visualized by Zeiss Cell Discoverer 7 using 10× magnification and acquisition of *Z*-stacks with 10 μm intervals between individual planes.

The hydrogels appeared to be suitable even for long-term culturing. Cells in hydrogels prepared from caged peptide showed no difference in terms of cell viability (Figure 4A) or cellular arrangement (cellular aggregate formation), compared to the hydrogel formed from the original EAK16-II peptide (Figure 4B).

## 4 Conclusions

Self-assembling peptides have a unique advantage as biomaterials due to their chemical variability. Unfortunately, despite their high biocompatibility and ease of modification—these peptides have poor solubility and handling, and as a result, their usage in biological systems is limited The formation of insoluble aggregates during synthesis or during chromatographic purification is a common problem of such peptides.

Our aim was to develop an approach to control the self-assembly property of these peptides. We hypothesized that the aggregation process could be inhibited by temporarily blocking critical amino acid side chains. Due to several biological and synthetic requirements (e.g., nontoxic metabolite, good solubility, and bulky), a photosensitive protecting group, pHP was chosen. However, pHP is incompatible with peptide synthesis conditions, a ^*t*^Bu-group-protected pHP derivative (Compound **6**) was developed. As a model self-assembling peptide, EAK16-II was chosen to test our theory. A caged derivative of this peptide (caged-EAK16-II) containing photolabile pHP protecting groups on two lysine side chains was synthesized.

The spectroscopic measurement confirmed that the developed caged peptide had good solubility and completely prevented aggregation in comparison with its noncaged, native form. Therefore, its purification and characterization could be carried out in the usual way as for peptides (e.g., RP-HPLC and LC–MS). RP-HPLC followed by photolysis demonstrated the release of the unprotected peptide and a nontoxic metabolite of the protecting group.

In vitro experiments with the caged peptide further verified our hypothesis. The self-assembling characteristic can be triggered by UV-light under controlled conditions at a suitable time and place. Hydrogels formed from the caged peptide via photolysis appeared to be similar to those obtained from native EAK16-II since both allowed the culturing and growth of A2058 human melanoma cells. No difference in cell viability and formation of cell aggregates was found between the caged and native EAK16-II-based hydrogels during in vitro measurements.

In conclusion, our caged peptide design provides a way to control the network formation of self-assembling peptides. Therefore, our approach opens up more opportunities for the use of such materials in the fields of biology and chemistry, such as controlled release models and immunization adjuvants.

## Supporting Information Available

HPLC, LC–MS, and ^1^H NMR spectra of different compounds as well as intensity data of DLS measurement (PDF)

## Abbreviations

HPAA: *p*-hydroxyphenylacetic acid
pHp: *p*-hydroxy-phenacyl
SPPS: solid phase peptide synthesis
^*t*^Bu: *tert*-butyl
