# Supplementary material for: Photoinduced Hydrogel-Forming Caged Peptides with Improved Solubility
Source: ACS Omega. 2024 Jan 30;9(6):6894–900. doi: 10.1021/acsomega.3c08289 (PMC10870261; doi:10.1021/acsomega.3c08289)
Supplement: Supplementary file 1 — ao3c08289_si_001.pdf [file ao3c08289_si_001.pdf]

# Supporting Information

## Photoinduced Hydrogel-Forming Caged Peptides with Improved Solubility

Kata N. Enyedi,[†,‡] BettinaBasa,[†,‡] GáborMező,[†,‡] EszterLajkó[*,§]

[†]Faculty of Science, Institute of Chemistry, Department of Organic Chemistry, Eötvös Loránd University, Pázmány Péter sétány 1/A, 1117 Budapest, Hungary

[‡]HUN-REN-ELTE Research Group of Peptide Chemistry, Eötvös Loránd University, Pázmány Péter sétány 1/A, 1117 Budapest, Hungary§

[§]Department of Genetics, Cell- and Immunobiology, Semmelweis University, Nagyvárad tér 4, 1089 Budapest, Hungary

*Email: lajko.eszter@med.semmelweis-univ.hu

*Compound 6*
Fmoc-L-Lys(pHP(tBu))-OH

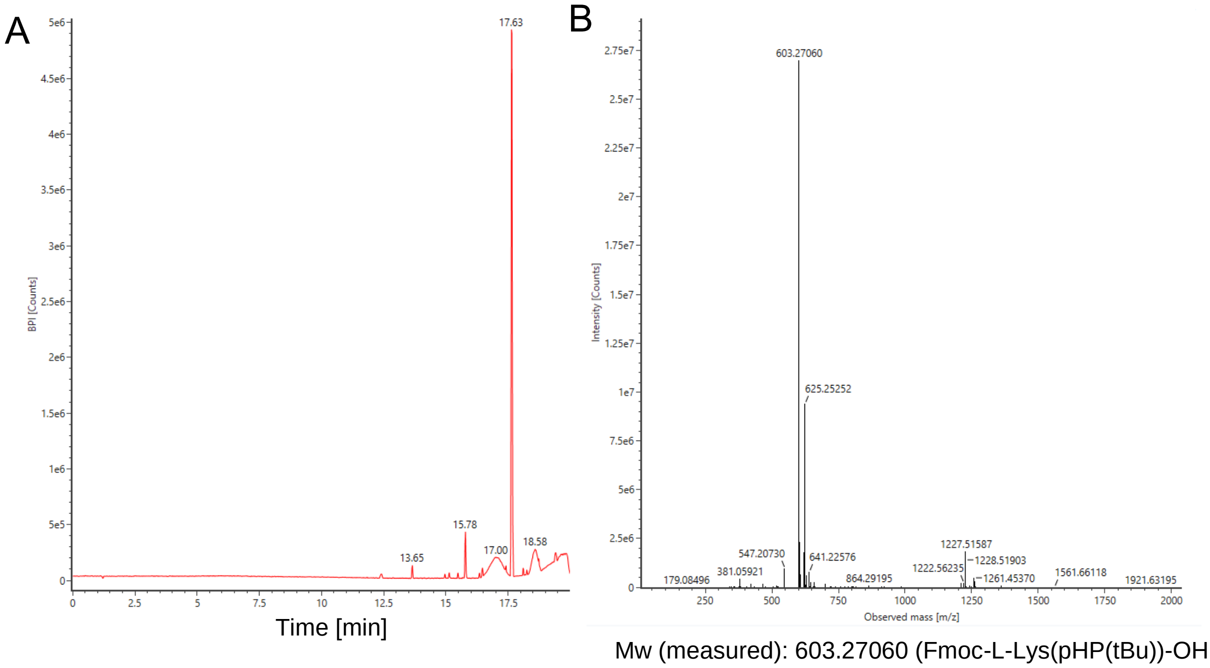


Mw (measured): 603.27060 (Fmoc-L-Lys(pHP(tBu))-OH, 1+, Δ 0.8 ppm)
547.20730 (-tBu, 1+, Δ 0.4 ppm)

*Figure S1 (A) represents the HPLC and (B) the MS spectra of Compound 6. Compound 6 was identified with LC-MS (Q-TOF instrument, Waters Corporation, Milford, MA, USA). Spectrum was acquired in the 50 – 2000 m/z range. Analytical RP-HPLC was performed on a KNAUER 2.1 S HPLC system (KNAUER, Bad Homburg, Germany) using a Macherey Nagel Nucleosil 100-5C18 column (250 mm x 4.6 mm; 5 µm). Linear gradient elution (0 min 0 % B; 5 min 0 % B; 50 min 90 % B) was applied at a flow rate of 1 mL/min. Peaks were detected at 220 nm.*

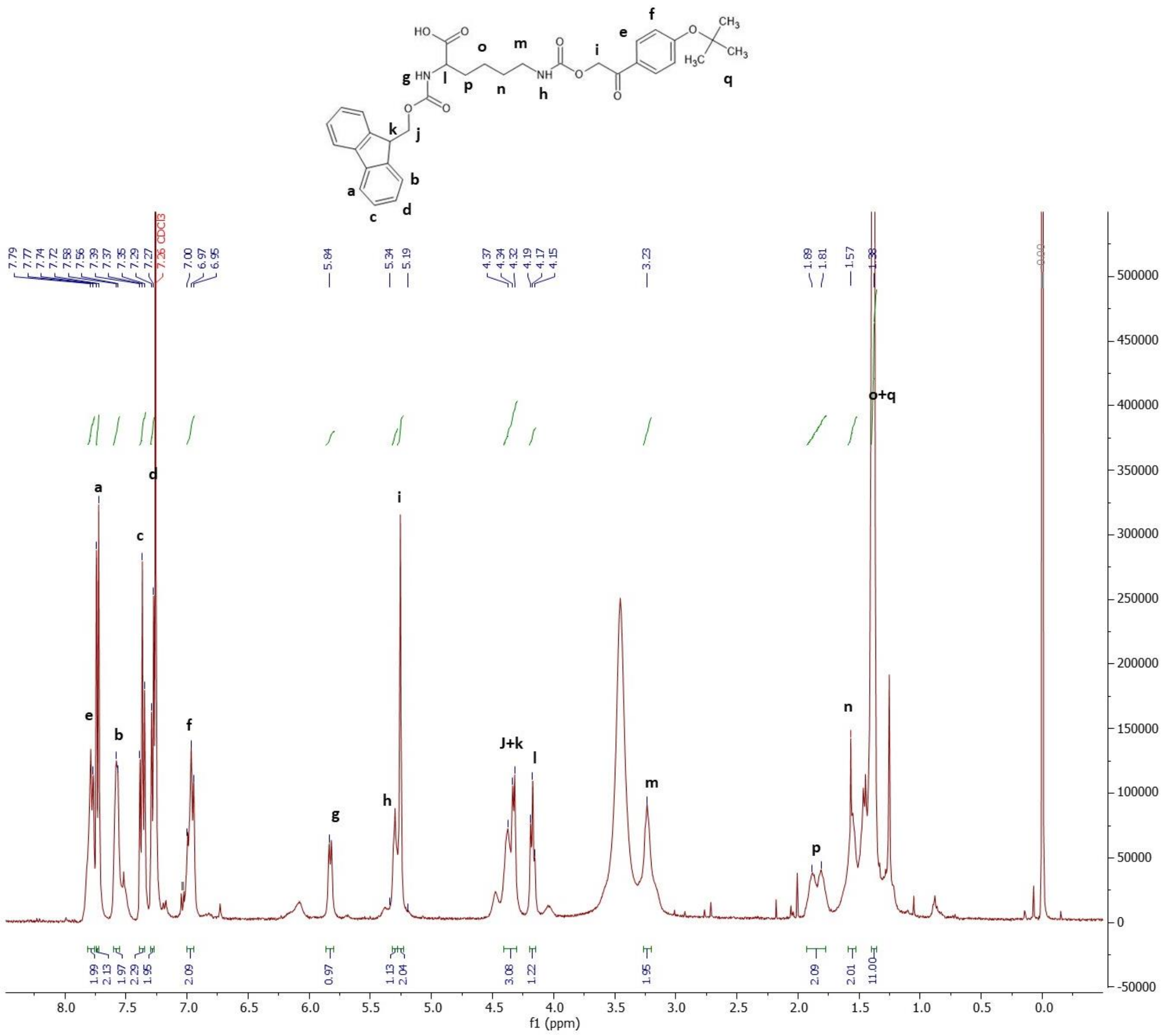

*Figure S2 $^{1}$H NMR spectrum of Compound 6. Compound 6 was identified with Bruker Ascend 400 spectrometer (Billerica, MA, USA).*

**Caged-EAK16-II**

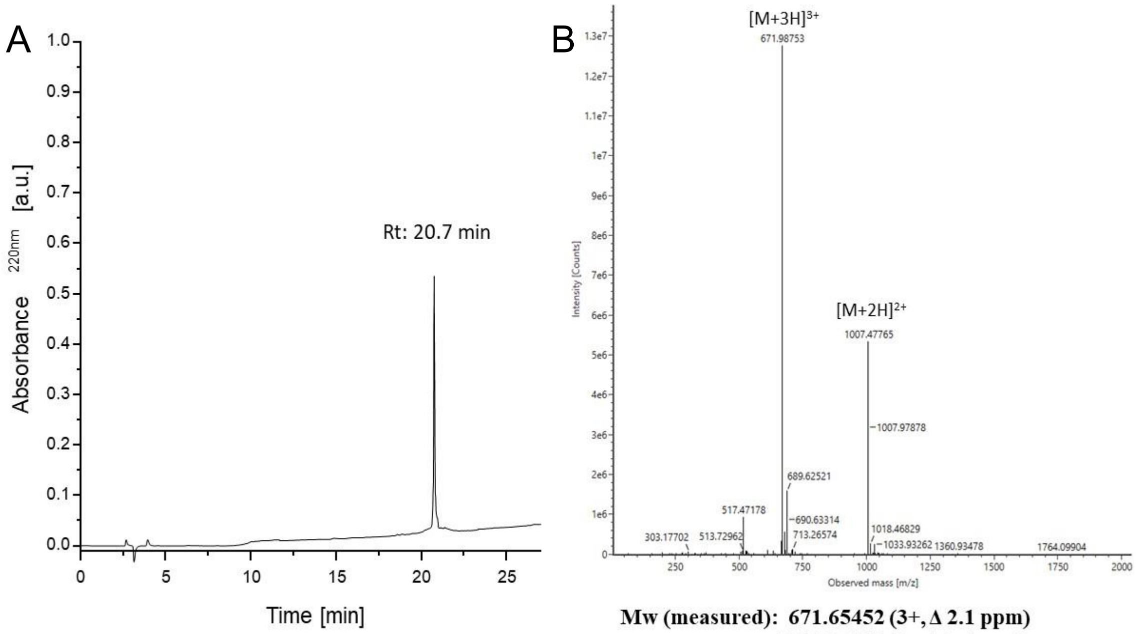


**Mw (measured): 671.65452 (3+, Δ 2.1 ppm)**
**1006.97658 (2+, Δ 0.6 ppm)**

*Figure S3 (A) represents the HPLC and (B) the MS spectra of caged-EAK16-II peptide. The peptide was identified with LC-MS (Q-TOF instrument, Waters Corporation, Milford, MA, USA). Spectrum was acquired in the 50 – 2000 m/z range. Analytical RP-HPLC was performed on a KNAUER 2.1 S HPLC system (KNAUER, Bad Homburg, Germany) using a Macherey Nagel Nucleosil 100-5C18 column (250 mm x 4.6 mm; 5 µm). Linear gradient elution (0 min 0 % B; 5 min 0 % B; 50 min 90 % B) was applied at a flow rate of 1 mL/min. Peaks were detected at 220 nm.*

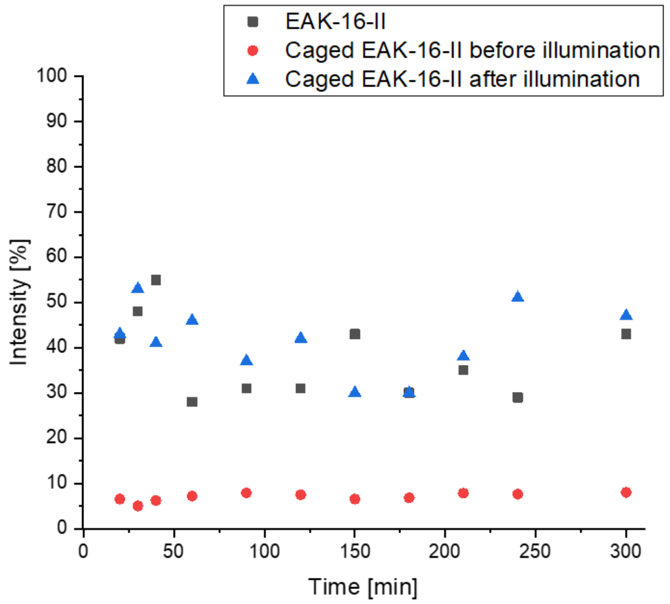


*Figure S4 The intensity of light scattering for native (non-protected) EAK16-II peptide and caged-EAK16-II (60 µM) by dynamic light scattering (DLS) measurements before and after illumination in PBS. The aggregation of the peptides was recorded on a Nanolab 3D instrument (LS Instruments, Switzerland) in PBS (phosphate buffered saline, pH 7.4, 1x) buffer.*
